# Severe Case of Cholestatic Hepatitis From Amoxicillin/Clavulanic Acid

**DOI:** 10.7759/cureus.25797

**Published:** 2022-06-09

**Authors:** Lokesh Goyal, Anirudh K Madabhushi, Mariam S Siddiqi, Santosh Kale, Deobrat C Mallick

**Affiliations:** 1 Hospital Medicine, CHRISTUS Spohn Hospital, Corpus Christi, USA; 2 Internal Medicine, University of the Incarnate Word School of Osteopathic Medicine, San Antonio, USA; 3 Internal Medicine, CHRISTUS Spohn Hospital, Corpus Christi, USA

**Keywords:** nausea and vomiting, abdominal pain, alkaline phosphatase (alp), drug-induced hyperbilirubinemia, hepatitis

## Abstract

The incidence of amoxicillin/clavulanate (Augmentin) induced liver injury is relatively low when compared to other medications. Amoxicillin/clavulanic acid is one of the most frequently prescribed antibiotics by physicians and is used to treat various bacterial infections. However, amoxicillin/clavulanate can cause severe side effects, usually gastrointestinal like nausea and vomiting, rash, and sometimes hematologic like thrombocytopenia. Here, we present a case report where a 63-year-old male treated for a dog bite with amoxicillin/clavulanate acid four weeks ago presents to the hospital with severe cholestatic hepatitis, nausea, and pruritis.

## Introduction

Amoxicillin/clavulanic acid is a commonly prescribed antibiotic used to treat respiratory and cutaneous infections such as bronchitis, sinusitis, community-acquired pneumonia, otitis media, and cellulitis. It is a combination of amoxicillin, a third-generation penicillin, and clavulanate, a beta-lactamase inhibitor. Amoxicillin works by inhibiting bacterial cell wall synthesis by binding to penicillin-binding proteins (PBPs), thereby inhibiting the final step of transpeptidation in peptidoglycan formation [1]. When clavulanate is added, it decreases bacterial resistance to penicillin by binding and inactivating beta-lactamases [1]. Amoxicillin itself has a rather narrow spectrum; however, adding clavulanate allows for wider coverage of gram-positive and gram-negative organisms [1]. The side effects of amoxicillin/clavulanic, such as gastrointestinal disturbances and rash, can be mild and self-limited. More severe adverse effects include hypersensitivity reactions, anaphylaxis, Stevens-Johnson syndrome, neutropenia, aplastic anemia, thrombocytopenic purpura, and drug-induced hepatitis [2]. Drug-induced hepatotoxicity is a major cause of acute liver failure in the US, and while the exact mechanism is still being studied, amoxicillin/clavulanic is one of the most common prescription drug causes, with hepatic injury occurring within three to four weeks from initial ingestion [3]. We present here a case report of a 63-year-old male patient who presented cholestatic hepatitis after completing high-dose amoxicillin/clavulanic in therapy several weeks prior.

## Case presentation

**Figure 1:**
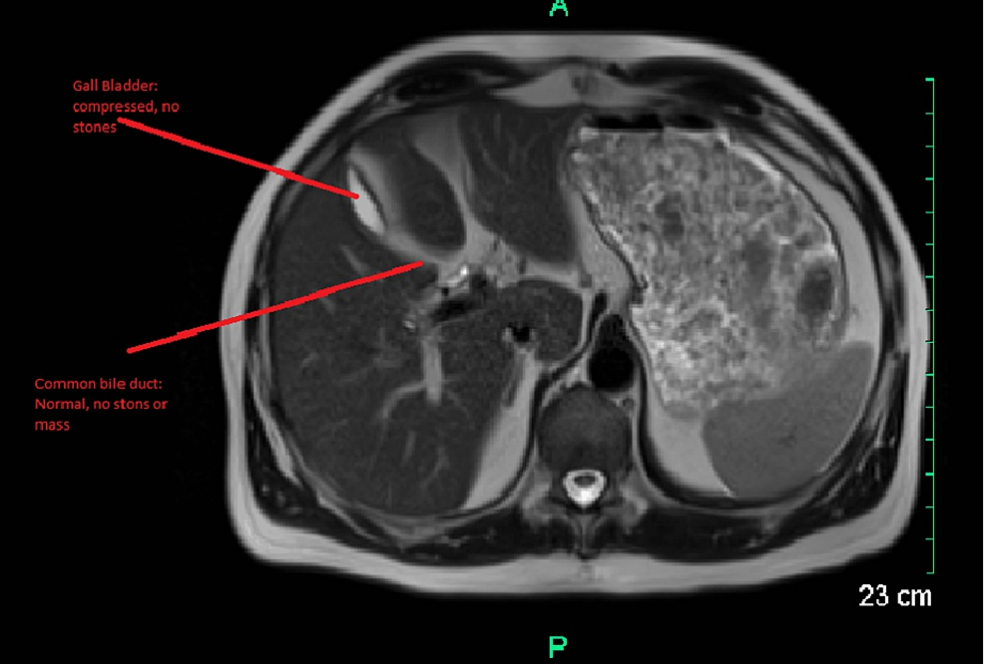
MRCP normal MRCP: magnetic resonance cholangiopancreatography

**Figure 2:**
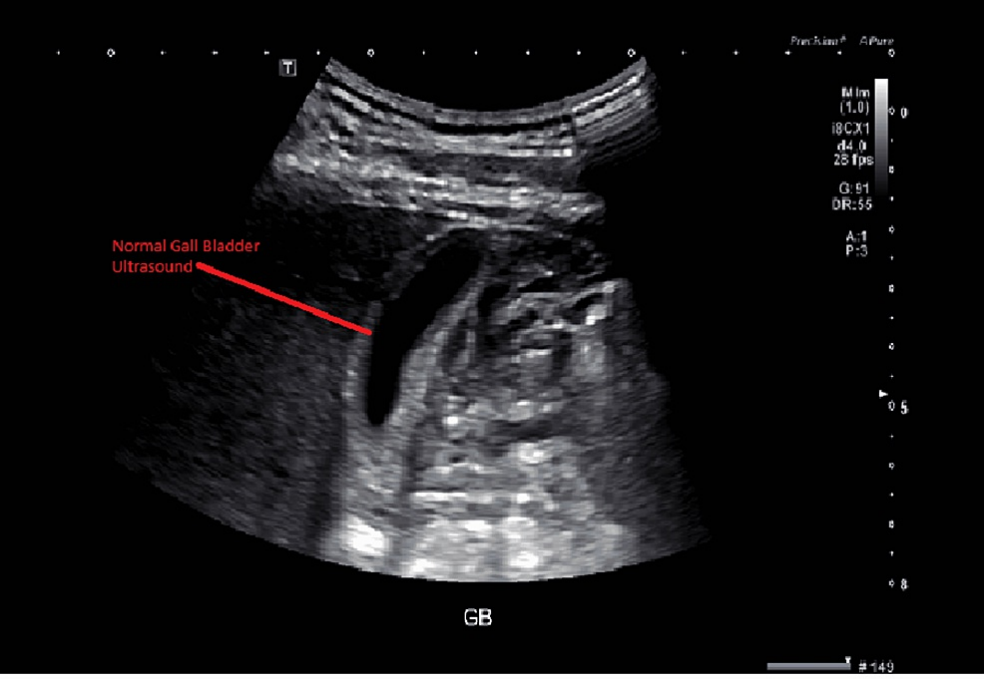
Gallbladder ultrasound: normal

The patient was a 63-year-old male with a past medical history of hyperlipidemia on pravastatin 40 mg daily who presented to the hospital with the chief complaint of itching going on for the past eight to 10 days. The patient also noticed that he had developed yellowness in his eyes and had some vague cramping in the stomach but no localized pain. He was evaluated in the emergency room and found to have elevated bilirubin of 29.1 mg/dl and alkaline phosphatase of 405 u/l. The patient had a completely normal CT abdomen pelvis with IV contrast and normal magnetic resonance cholangiopancreatography (MRCP) and right upper quadrant ultrasound (Figures 1-2).

The hepatitis panel was also negative. The patient stated that he drank one to two beers once a month. He also mentioned that he was bitten by a dog two weeks ago and was prescribed amoxicillin/clavulanate 875 mg p.o. twice daily for 14 days. He denied any history of gastrointestinal issues and denied trouble swallowing, heartburn, indigestion, nausea, or vomiting.

On physical exam, we noticed that the patient's skin color was completely yellow with scleral icterus. The patient otherwise moved all extremities. His abdomen was soft and non-tender to palpate. No lymphadenopathy in the neck, axilla, or groin was noticed.

Gastroenterology was consulted for this patient, and pravastatin was stopped. The patient had already stopped taking amoxicillin/clavulanate four weeks ago before coming to the hospital. The patient was placed on IV fluids, metoclopramide for nausea, and cholestyramine for pruritis. The gastroenterologists recommended monitoring the patient's liver enzymes and bilirubin for 48 hours. The gastroenterologist recommended that if the liver enzymes get any worse, the patient may need steroids; however, fortunately, in this case, the patient's liver enzymes and bilirubin started trending down in the next 48 hours (Table 1).

**Table 1:** Blood tests

Lab results	Reference Range	Day 1	Day 5	Day 10	Day 14
Total Bilirubin	<1.40 mg/dL	29.1 mg/dl	18.1 mg/dl	11.6 mg/dl	6.9 mg/dl
Alanine Transaminase	<41 U/L	256 U/L	198 U/L	144 U/L	120 U/L
Aspartate Transaminase	<40 U/L	244 U/L	212 U/L	129 U/L	111 U/L
Alkaline Phosphatase	40-130 U/L	405 U/L	350 U/L	278 u/L	240 U/L
Lipase	13-60 U/L	18 u/L			
Complete Blood Count	Normal	Normal			
Blood Alcohol Level	Negative	Negative			
Urine Drug Screen	Negative	Negative			

The patient's bilirubin went down from 29.1 mg/dL to 18.1 to 6.9 mg/dL in two weeks. Also, the patient's liver enzymes trended down from alanine transaminase (ALT) of 256 U/L to 120 U/L. Also, alkaline phosphatase (ALP) went down from 405 U/L to 240 U/L. The patient was then discharged home and educated to follow up with his primary care physician in one week.

## Discussion

Amoxicillin/clavulanate-induced hepatic damage has an incidence of 1.7 compared to 0.3 in 10,000 prescriptions for the use of amoxicillin alone and is mainly cholestatic lesions, but cases of hepatocellular, mixed hepatocellular-cholestatic and granulomatous lesions have been reported [3-5]. The onset of hepatotoxicity usually occurs between a few days to eight to 10 weeks and rarely leads to death when underlying comorbidities, such as cirrhosis, are present [2-3]. The mechanism of injury is not well understood but is thought to be immunoallergic in nature [2]. It has also been shown that amoxicillin/clavulanate-induced liver injury is associated with human leukocyte antigen (HLA) DRB1*1501-DRB5*0101-DQB1*0602 haplotypes [3,6], and there is an idiosyncrasy in HLA Class II antigens that play a role in this pathological process [1-3,7]. Histologically, we would typically see portal or periportal lymphocytic inflammation, centrilobular or panlobular cholestasis, along with eosinophils and neutrophils (3,5-6). Signs and symptoms of amoxicillin/clavulanate-induced hepatoxicity include signs of hypersensitivity such as a fever, rash, and eosinophilia [2-3,8], as well as cholestatic symptoms, including jaundice and fecal acholia [3,9]. Risk factors for amoxicillin/clavulanate hepatotoxicity include male sex, alcohol consumption, multiple courses of the drug, over the age of 55, and use of other hepatotoxic substances [1,3,8]. The duration of antibiotic therapy and older age seem to be the main key factors in the development of a cholestatic injury pattern [6,9]. It has also been hypothesized that the amoxicillin/clavulanate-induced hepatotoxicity seen mostly in older people could be attributed to a slower rate of elimination, which causes prolonged exposure in the bile duct cells and leads to immune response [9].

Treatment is mainly supportive and should include fluids, anti-emetics, and analgesics to improve hydration status and pruritus [3]. Although corticosteroids have been used as treatment, there is no proof of efficacy [2]. Ursodiol and cholestyramine can be used for symptomatic treatment but have not been shown to increase recovery time [2-3,5-6].

## Conclusions

This case report highlights the importance of taking an accurate patient history and knowing the side effects of the most commonly prescribed antibiotic in our population today. This report will help our medical professionals accurately identify and treat these side effects without performing unnecessary procedures/imaging and help in reducing the healthcare cost. A case report such as this one brings the attention of its readers to the rare side effects of this commonly prescribed antibiotic.
